# Transient Unilateral Blepharoptosis Following Upper Blepharoplasty: A Case Report and Considerations for Local Anesthetic Use

**DOI:** 10.7759/cureus.79817

**Published:** 2025-02-28

**Authors:** Georgios E Papanikolaou, Konstantina Bouranta, Thomas Iraklis Smiris, Dimitrios N Varvarousis

**Affiliations:** 1 Plastic Surgery, Private Practice, Ioannina, GRC; 2 Medical School, University of Ioannina, Ioannina, GRC; 3 Laboratory of Anatomy-Histology-Embryology, University of Ioannina, Ioannina, GRC

**Keywords:** blepharoptosis, epinephrine toxicity, lidocaine toxicity, local anesthetic, upper blepharoplasty, upper eyelid edema

## Abstract

Upper blepharoplasty is a common aesthetic procedure aimed to address dermatochalasis of the eyelid and improve visual acuity. During the postoperative period, patients may experience complications that are usually minor and transient. However, these complications may adversely affect the healing process, delaying the desired result. Accordingly, we present a case of a 57-year-old female with late and transient unilateral blepharoptosis after cosmetic upper blepharoplasty. Preoperative examination revealed the presence of bilateral upper eyelid dermatochalasis and mild prolapse of the nasal fat pad, normal function of the orbicularis oculi, levator palpebrae superioris and frontalis muscles, without evidence of ptosis. Medical history included hyperlipidemia without other significant comorbidities. We hypothesize that the additional local anesthetic injection at the affected eyelid in combination with the persistent edema could have caused the development of ptosis. Therefore, we emphasize the careful use of local anesthetic solution in cases of blepharoplasty and the detailed preoperative examination of the patients to exclude the presence of preexisting blepharoptosis. Moreover, we propose an intraoperative strategy to reduce the possibility of injury of the levator palpebrae superioris muscle and aponeurosis, and therefore to improve postoperative recovery in upper blepharoplasty.

## Introduction

Upper blepharoplasty is one of the most popular cosmetic procedures, aimed to effectively address dermatochalasis and excess of the central and nasal fat pads, improving at the same time the visual field [1,2]. The postoperative complications of upper blepharoplasty can be categorized based on the timeframe of appearance as early (within the first week), intermediate (weeks one through six), and late (after six weeks) [3]. Although common complications such as edema, hematoma, and ptosis are usually minor and transient, they can delay wound healing and adversely affect the final functional and aesthetic result [4,5].

Particularly, blepharoptosis can occur after upper blepharoplasty, but there is no evidence in the literature about its incidence [3,6]. The anatomic structures that play an important role in the pathogenesis of the ptosis is the orbicularis oculi muscle, which function is to close the eyelids, the levator palpebrae superioris (LPS) muscle and its aponeurosis, which acts as the main retractor of the upper eyelid, and the Muller’s muscle, which is innervated by the sympathetic autonomous system and acts as an accessory upper lid elevator [1]. The etiologic factors that may induce postoperative blepharoptosis are mainly edema, hematoma, and mechanical trauma directly to the LPS muscle and aponeurosis [3,5,6]. Therefore, it is important to refine our surgical techniques to minimize anatomic damage at the upper eyelid and to reduce patients’ downtime.

We report an uncommon case of long-lasting unilateral blepharoptosis after cosmetic upper blepharoplasty, hypothesize that it is due to an excessive local anesthetic injection and prolonged postoperative edema, emphasizing the importance of cautious use of anesthetic technique and the necessity of a thorough preoperative examination to diagnose a preexisting blepharoptosis. Additionally, we aimed to propose an intraoperative strategy to reduce the possibility of injury of LPS muscle and aponeurosis, and therefore to improve patients’ postoperative recovery after upper blepharoplasty.

## Case presentation

The study was conducted in accordance with the Declaration of Helsinki and relevant ethical national guidelines. Written informed consent was obtained from the patient for the publication of this case report and the use of the accompanying images and clinical data. This is a case report in which no research has been done.

**Figure 1:**
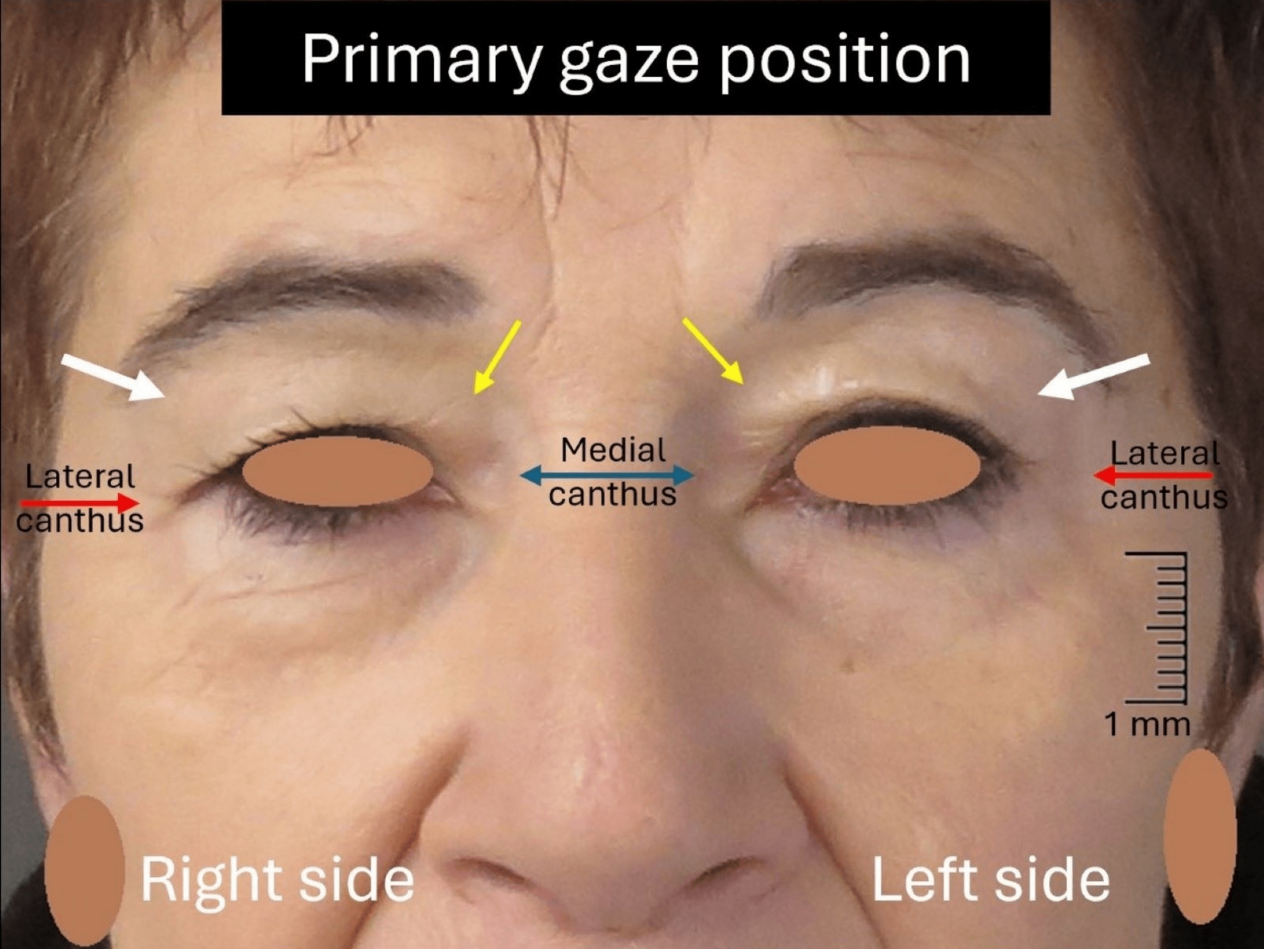
Preoperative image of the patient Notice the significant dermatochalasis (white arrows) at the upper eyelids covering the eyelid margin at the lateral canthus (red arrows) and the prolapse of the nasal fat pad (yellow arrows) over the medial canthus (blue double arrow). There is no evidence of ptosis.

A 57-year-old Caucasian female presented with the complaint of drooping upper eyelids associated with visual restriction affecting negatively the activities of daily living. Physical examination revealed the presence of bilateral excess upper eyelid skin and mild prolapse of the nasal fat pad, normal function of the orbicularis oculi, LPS, and frontalis muscles, without evidence of ptosis (Figure 1).

**Figure 2:**
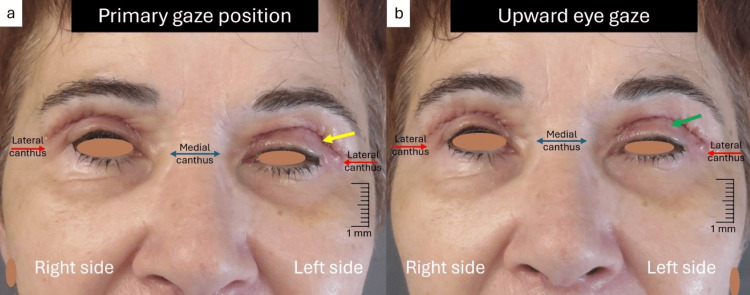
One week postoperative view a) Notice the severe edema (yellow arrow) at the left upper eyelid with evidence of moderate blepharoptosis; (b) Complete absence of LPS muscle motility in the upward eye gaze (green arrow) at the left upper eyelid LPS: levator palpebrae superioris

Therefore, we decided to proceed to the upper blepharoplasty under local anesthesia. The local anesthetic solution used was a preparation of lidocaine hydrochloride 2% (20 mg/ml) and epinephrine 1:100.000 (0,01 mg/ml). We injected about 2 ml of the anesthetic solution per side using a 30-gauge and 13 mm-long sharp needle. Initially, an ellipse of redundant skin was excised, with preservation of the orbicularis oculi muscle. Afterward, the prolapsed nasal fat was removed, and meticulous hemostasis was achieved. However, due to the patient's persistent pain at the left side, we injected an additional 0.5 ml of the anesthetic solution, mainly near the lower edge of the upper eyelid skin incision. Skin closure was achieved with a running 6-0 prolene suture. The immediate postoperative course was uneventful. One week later, we removed the stitches, but despite the significant reduction of the edema and ecchymosis, we noticed moderate blepharoptosis at the left side with a complete absence of LPS muscle function (Figures 2a, 2b).

**Figure 3:**
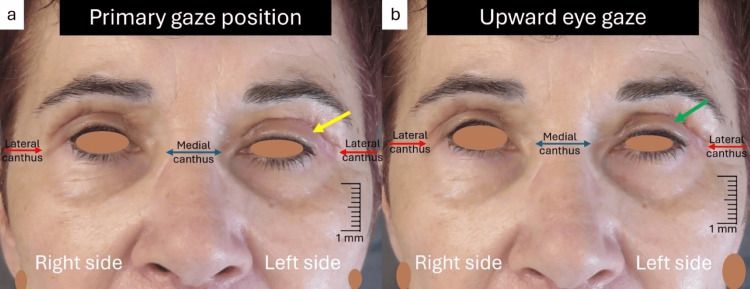
Four weeks postoperative view (a) The ptosis is still evident at the left side, with persistent edema (yellow arrow); (b) Light improvement of the LPS muscle motility (green arrow) in upward eye gaze LPS: levator palpebrae superioris

We advised the patient to continue with mild cold compresses and light massages of the affected area, and to perform a dynamic upward movement of the eyelids. However, four weeks later, the ptosis was still evident at the left side with persistent edema and light improvement of the LPS muscle movement in upward eye gaze (Figures 3a, 3b).

**Figure 4:**
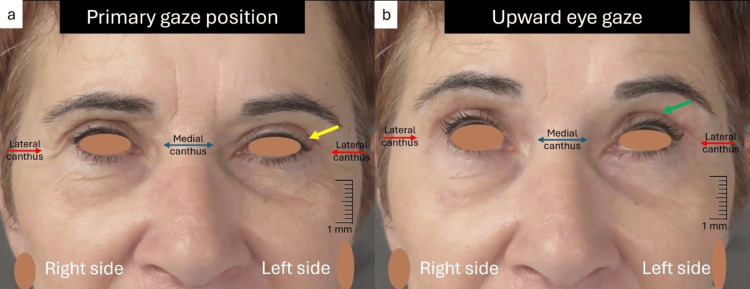
Seven weeks postoperative view (a) Complete resolution of the edema at the left upper eyelid (yellow arrow); (b)  Compete recovery of the LPS muscle motility at the left upper eyelid (green arrow) LPS: levator palpebrae superioris

**Figure 5:**
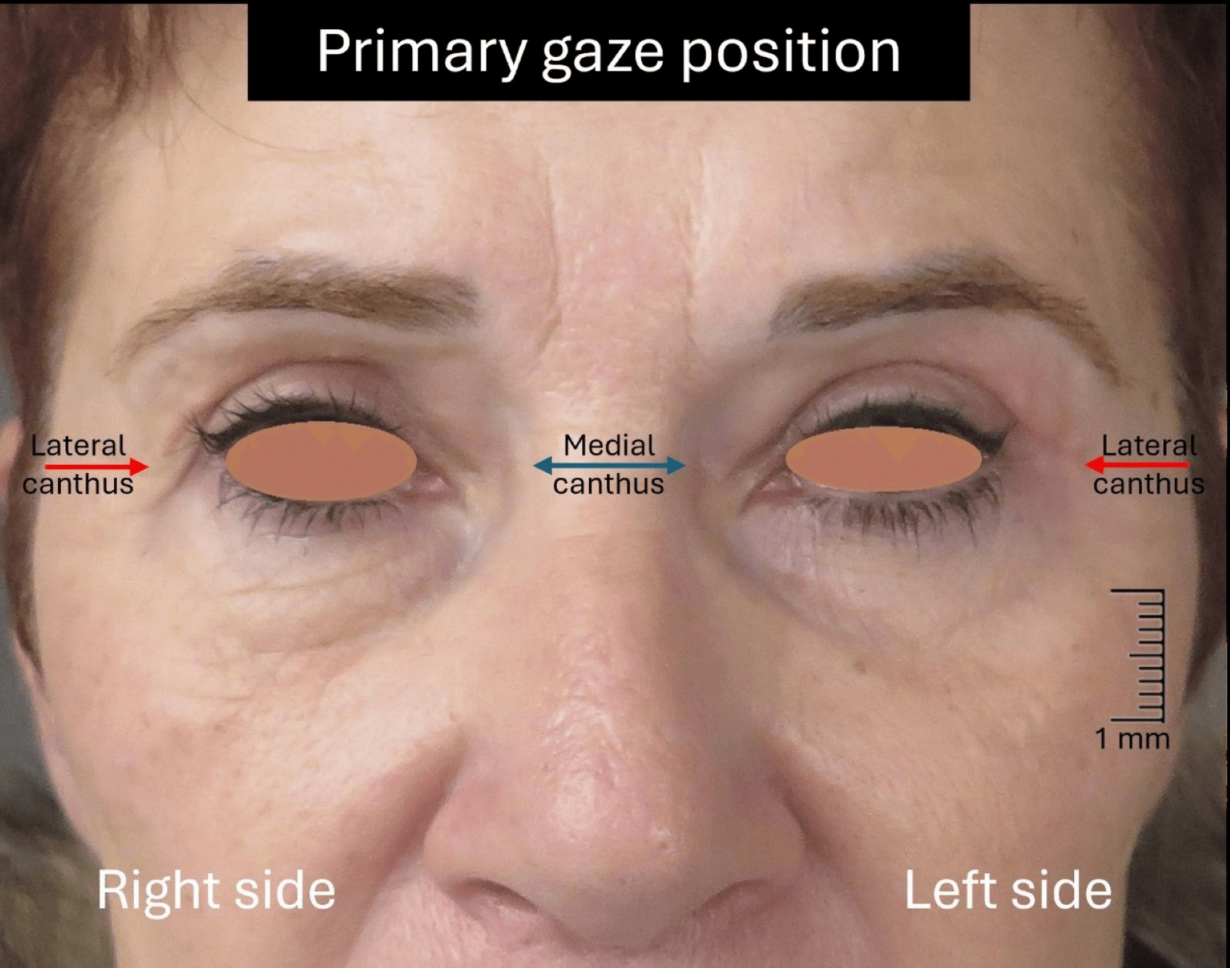
Postoperative view after four months, with excellent aesthetic and functional outcome

During the next weeks further improvement of the LPS muscle function was observed, and the edema resolved, with complete recovery of the ptosis after seven weeks (Figures 4a, 4b). The patient achieved a satisfactory aesthetic and functional outcome (Figure 5).

## Discussion

The preoperative examination in patients seeking cosmetic upper blepharoplasty must assess both the presence of an already established ptosis (congenital or acquired) and the extension of eyelid dermatochalasis that may cover an altered marginal reflex distance [1,4]. Interestingly, preoperative analysis of sexual dimorphism in cranial dimension may help in understanding anatomic variations, such as the presence of ptosis, which will ultimately allow for safer and personalized surgical techniques [7]. Once the preoperative ptosis is excluded, we must consider possible intraoperative parameters that can lead to upper eyelid ptosis after the operation. The most common causes of postoperative ptosis include edema, ecchymosis/hematoma causing attenuation of the LS muscle and aponeurosis, inadvertently suturing of the orbital septum to the LPS muscle or aponeurosis, and direct trauma to the levator aponeurosis with the surgical instruments and/or the electrocautery in case of accidentally opening of the orbital septum [3,5,6].

In the current case, we used a local anesthetic solution containing lidocaine hydrochloride 2% with epinephrine 1:100.000. The injection of different local anesthetics can induce myotoxicity, and the muscular damage is greater with high concentrations and prolonged anesthetic duration [8]. In the present case, the LPS muscle recovered completely after seven weeks, given that the muscle injury is usually reversible, and full recovery is achieved within four weeks [8]. Accordingly, we hypothesize that lidocaine may have caused a transient paralysis of the LPS muscle and Muller’s muscle. Moreover, the addition of epinephrine can increase the lidocaine-induced muscle injury [8-10], even if epinephrine could stimulate the Muller’s muscle activity [11].

Further mechanisms that potentially could have caused the ptosis in our patient are the additional volume of local anesthetic reinjected in the left side due to persistent pain and the site of the reinjection at the inferior edge of the initial blepharoplasty skin incision. The use of the high volume of local anesthetic may induce mechanical blepharoptosis [11], while a deep injection at the inferior part of the operative field may damage the levator aponeurosis, as this area is the insertion of the levator aponeurosis to the orbicularis oculi muscle and skin [12]. Similarly, Hwang et al. recommend avoiding local anesthetic injection into the distal third of the LPS muscle at the level of the orbital rim, because at this level the terminal branches of the oculomotor nerve enter the LPS muscle, where they are more exposed and vulnerable to iatrogenic damage that could lead to transient or permanent blepharoptosis [13]. 

Interestingly, we noticed a persistent and severe edema at the left upper eyelid four weeks after the operation. Usually, most of the swelling is resolved during the early postoperative period, without leaving any complications. We assume that prolonged edema could have induced attenuation of the levator aponeurosis, causing a temporary restriction of the LPS muscle function. Recent studies have demonstrated that the use of buffered combinations of local anesthetics in periocular operations, such as bicarbonate-buffered lidocaine with epinephrine and bicarbonate-buffered lidocaine in a 3:1 ratio, can produce significantly lower pain, operative bleeding, and postoperative swelling [14,15].

For the injection of the local anesthetic, we used a 30-gauge and 13 mm-long sharp needle. Although this is a common practice, there is current evidence that the use of very small (nanosoft, integral array of three microneedles; each microneedle 0.6 mm in length) and blunt needles may reduce postoperative edema, ecchymosis, and hematoma rates, with faster recovery after upper blepharoplasty [16,17].

The early treatment of postoperative blepharoptosis is conservative, including cool compresses, light massage, and active movements of the upper eyelid, where persistent edema can be treated with oral diuretics and steroids [2]. In most of the cases, eyelid dysfunction is temporary and resolves within three months. However, the presence of permanent ptosis is indicative of levator dehiscence, and surgical correction must be performed, involving direct repair of the levator injury [5].

To our knowledge, the present case is one of the best documented cases of transient blepharoptosis after cosmetic blepharoplasty, although it is not that rare. An import limitation of our study is the lack of objective ptosis measurements, restricting its diagnostic and therapeutic utility. However, different studies have shown the infrequent occurrence of transient or permanent blepharoptosis in ophthalmic and periocular operations, without quantitative measurements [6,9,18-20].

Based on our experience and the reported literature evidence, we suggest using a buffered-based local anesthetic solution, placing the injection of the local anesthetic superficially at the central part of the upper eyelid, using a small and eventually blunt needle, as well as a low volume of the anesthetic solution to avoid injury of the LPS muscle and aponeurosis, and reduce patients’ downtime. Moreover, the patient must be assessed for the existence of preoperative blepharoptosis and should be informed in detail about the risk of postoperative ptosis and the relevant treatment options.

## Conclusions

Postoperative blepharoptosis after aesthetic upper blepharoplasty is usually temporary and is referred to more as a symptom than a complication. However, surgeons must be aware of the potential myotoxicity that could occur with the administration of the local anesthetic solution. Similarly, we emphasize the importance of considering the use of the local anesthetic and the presence of persistent edema as potential causal factors in the differential diagnosis of postoperative blepharoptosis. Moreover, we highlight the importance of a thorough preoperative examination for the diagnosis of preexisting blepharoptosis, and we propose an intraoperative strategy to avoid possible injury of the LPS muscle and aponeurosis, and therefore to reduce patients’ downtime in upper blepharoplasty.
